# Intratumoral Administration of Holmium-166 Acetylacetonate Microspheres: Antitumor Efficacy and Feasibility of Multimodality Imaging in Renal Cancer

**DOI:** 10.1371/journal.pone.0052178

**Published:** 2013-01-08

**Authors:** Wouter Bult, Stephanie G. C. Kroeze, Mattijs Elschot, Peter R. Seevinck, Freek J. Beekman, Hugo W. A. M. de Jong, Donald R. A. Uges, Jos G. W. Kosterink, Peter R. Luijten, Wim E. Hennink, Alfred D. van het Schip, J. L. H. Ruud Bosch, J. Frank W. Nijsen, Judith J. M. Jans

**Affiliations:** 1 Imaging Division, Department of Radiology and Nuclear Medicine, University Medical Center Utrecht, Utrecht, The Netherlands; 2 Department of Hospital and Clinical Pharmacy, University Medical Center Groningen, Groningen, The Netherlands; 3 Department of Urology, University Medical Center Utrecht, Utrecht, The Netherlands; 4 Laboratory of Experimental Oncology, University Medical Center Utrecht, Utrecht, The Netherlands; 5 Image Sciences Institute, University Medical Center Utrecht, Utrecht, The Netherlands; 6 Milabs, Utrecht, The Netherlands; 7 Section Radiation Detection & Medical Imaging, Faculty of Applied Sciences, Delft University of Technology, Delft, The Netherlands; 8 Department of Pharmaceutics, Utrecht Institute for Pharmaceutical Sciences, Utrecht University, Utrecht, The Netherlands; The University of Chicago, United States of America

## Abstract

**Purpose:**

The increasing incidence of small renal tumors in an aging population with comorbidities has stimulated the development of minimally invasive treatments. This study aimed to assess the efficacy and demonstrate feasibility of multimodality imaging of intratumoral administration of holmium-166 microspheres (^166^HoAcAcMS). This new technique locally ablates renal tumors through high-energy beta particles, while the gamma rays allow for nuclear imaging and the paramagnetism of holmium allows for MRI.

**Methods:**

^166^HoAcAcMS were administered intratumorally in orthotopic renal tumors (Balb/C mice). Post administration CT, SPECT and MRI was performed. At several time points (2 h, 1, 2, 3, 7 and 14 days) after MS administration, tumors were measured and histologically analyzed. Holmium accumulation in organs was measured using inductively coupled plasma mass spectrometry.

**Results:**

^166^HoAcAcMS were successfully administered to tumor bearing mice. A striking near-complete tumor-control was observed in ^166^HoAcAcMS treated mice (0.10±0.01 cm^3^ vs. 4.15±0.3 cm^3^ for control tumors). Focal necrosis and inflammation was present from 24 h following treatment. Renal parenchyma outside the radiated region showed no histological alterations. Post administration CT, MRI and SPECT imaging revealed clear deposits of ^166^HoAcAcMS in the kidney.

**Conclusions:**

Intratumorally administered ^166^HoAcAcMS has great potential as a new local treatment of renal tumors for surgically unfit patients. In addition to strong cancer control, it provides powerful multimodality imaging opportunities.

## Introduction

Kidney cancer accounts for approximately 3% of all cancers. With a world-wide incidence of 208,000 new cases and a mortality of 102,000 patients each year, it is one of the most lethal genitourinary malignancies [1]. In recent years a dramatic increase of incidentally detected small renal tumors has occurred, mainly due to more widespread use of non-invasive imaging techniques. These tumors are frequently found in patients who are at surgical risk due to factors such as advanced age and comorbidities [2]. It has been shown that not every patient will benefit from surgical resection of the tumor and that it is better to avoid surgically-induced morbidity in several cases [3], [4], [5]. The recommended treatment for small renal tumors is nephron-sparing surgery [6], but for patients for whom surgical resection is considered inappropriate, minimally invasive techniques are also being developed [7], [8]. Although historically external beam radiotherapy (EBRT) has not been effective for treatment of renal tumors due to breathing-related movement of the kidneys and radiosensitivity of adjacent tissue, new radiation methods may provide a successful alternative [9]. An important new development to circumvent the radiosensitivity issues of healthy tissue around the tumor is the selective local administration of radioactive sources to the tumor [10], [11]. An example of this selective local administration is transcatheter radioembolization with yttrium-90 (^90^Y) resin and glass microspheres (MS), which has shown promising results in treatment of unresectable hepatic metastases [12], [13], [14]. Possibly, intratumoral injection might be more effective than intra-arterial injection [15]. In this paper, a novel local ablation technique is presented using small (10–15 µm) holmium-166 acetylacetonate microspheres (^166^HoAcAcMS) with a high holmium load [16]. ^166^Ho emits both high-energy beta particles (E_βmax_ 1.77 and 1.85 MeV, maximum tissue penetration 8 mm, mean tissue penetration 2.5 mm) and gamma rays (0.081 MeV) that allow for nuclear imaging and has a half-life of 26.8 h [17]. Moreover, non-radioactive holmium-165 can be visualized by CT and MRI, due to its high mass attenuation coefficient and its paramagnetic properties, respectively [18]. These imaging opportunities offer many advantages such as visualizing the distribution of holmium microspheres inter- and post-treatment for direct therapy evaluation and follow-up, as well as MRI-based absorbed dose estimation [19]. This study provides a proof of principle for intratumoral administration of ^166^HoAcAcMS as a novel treatment strategy for kidney cancer in patients not eligible for surgery, and demonstrates the elegant *in vivo* multimodality imaging necessary for treatment guidance and monitoring.

## Materials and Methods

### Cell culture

Balb/C renal carcinoma cells (Renca, National Cancer Institute, USA) were maintained at 37°C and 5% CO_2_ in Dulbecco's Modified Eagle's Medium (Lonza, the Netherlands) supplemented with 10% fetal bovine serum (Lonza, the Netherlands), 1% penicillin/streptomycin and 1% glutamine. To prepare for *in vivo* transplantation, cells were trypsinized, washed and kept on ice until transplantation.

### Ethics Statement

All experimental protocols were conducted in agreement with the Netherlands Experiments on Animals Act and the European convention guidelines, and accepted by the Animal Experiments Committee Utrecht, the Netherlands (2009.III.01.003).

### Animal experiments

Male Balb/C mice (Charles River, the Netherlands) aged 9–11 weeks were used for experiments. All surgical procedures were performed under isoflurane anesthesia (IsoFlo, Abbott Animal Health, the Netherlands). To provide analgesia each mouse received 3 µg buprenorfine subcutaneously (Buprecare, AST Pharma, the Netherlands) prior to, and 24 h post surgery.

### Orthotopic kidney tumor model

In 60 mice, the left kidney was exposed through a flank incision and a Renca tumor cube of 2 mm diameter was transplanted under the renal capsule. Renal tumors were allowed to grow for 1 week, while well being of mice was followed by measuring body weight and scoring physical appearance.

### Holmium acetylacetonate MS

HoAcAcMS were prepared as described by Bult et al [17]. In short, HoAcAcMS were prepared using a solvent evaporation technique. Holmium acetylacetonate was dissolved in chloroform (HPLC grade, Sigma Aldrich, The Netherlands), followed by emulsification in water containing 2% polyvinylalcohol (average MW 30,000–70,000 Da, Sigma Aldrich, The Netherlands) as an emulsifier. After evaporation of chloroform under continuous stirring, HoAcAcMS were harvested and washed with water before sieving to obtain the preferred particle size (10–15 um). The sieve fractions were dried at 40°C. The particle size was determined using a Coulter Counter, and the holmium content was determined using the complexometric titration as described by Zielhuis et al. [20]. Sixty mg HoAcAcMS was transferred to a high-density poly-ethylene vial (Posthumus Plastics, the Netherlands), and neutron irradiated for one hour with a thermal neutron flux of 5×10^12^ n cm^−2^ s^−1^, using the pneumatic rabbit system in a nuclear reactor (Reactor Institute Delft, the Netherlands) to render the MS radioactive. Radionuclidic purity was determined batchwise after neutron activation to ^166^HoAcAcMS. Analysis was done by gamma spectroscopy using a high-purity germanium detector and GammaVision software. The gamma spectrum obtained was compared to reference data for holmium-166 from ICRP 38 (ICRP, 1983. Radionuclide Transformations – Energy and Intensity of Emissions. ICRP Publication 38. Ann. ICRP 11–13). For the batch of ^165^HoAcAcMS used in this study no peaks other than from holmium-166 were detectable after neutron activation.

To assess the MS integrity after neutron irradiation particle size was determined using a coulter counter (Multisizer 3, Beckman Coulter, the Netherlands), performing light microscopy and electron microscopy (Phenom, Phenom B.V., the Netherlands). The size of the HoAcAcMS used range from 10–15 µm. Earlier in vitro and in vivo stability studies conducted with these HoAcAcMS before and after neutron irradiation have been described in detail by Bult et al. [21]. Briefly, in vitro release of holmium was assessed after incubation at 37°C in phosphate buffer for 24 hours up to 180 days showing <0.3% and <0.5% free holmium, respectively. HoAcAcMS before and after neutron irradiation were also administered intratumorally in VX2 tumor-bearing rabbits. No holmium was detected in the faeces, urine, femur and blood of the rabbits, and histological examination of the tumor revealed clusters of intact microspheres amidst necrotic tissue after thirty days. These studies have shown that the radiochemical purity and stability of the HoAcAcMS is high and diffusion throughout the tissue is not expected.

### Administration of microspheres

Radioactive ^166^HoAcAcMS (600 MBq) were suspended in 1.2 ml of an aqueous poloxamer F68 solution (Pluronic® solution, 2% w/v), and 50 µl was taken up in 29 G insulin syringes (Becton Dickinson Ultra Fine, the Netherlands). The activity was measured using a dose calibrator (VDC-404, Veenstra Instruments, the Netherlands). Prior to administration, the syringes were placed in an acrylic glass cylinder to limit the dose to the hands. The syringe was agitated vigorously to obtain a homogenous suspension and 10 µl of the ^166^HoAcAcMS (approximately 5 MBq or 500 µg MS) suspension was administered intratumorally via an open surgical approach (n = 4–5/group). After administration the syringes were measured in a dose calibrator to calculate the dose administered to the tumors. Control mice (n = 3–7/group) received intratumoral administration of 0.9% NaCl. At 2 hours, 1, 2, 3, 7 and 14 days after administration of MS mice were sacrificed by cervical dislocation.

### Imaging

Immediately following administration of ^166^HoAcAcMS, anesthetized mice were placed in a small animal CT (U-CT, MILabs, the Netherlands). Images were acquired at a tube voltage of 45 kV, a tube current of 350 mAs and a voxel size of 83 µm (isotropic). Two mice underwent multimodality imaging. Single Photon Emission Computed Tomography imaging (SPECT) was performed on a U-SPECT system (MILabs, the Netherlands) with a general purpose mouse collimator (75 focussing 0.6 mm diameter pinholes) to assess the ^166^HoAcAcMS distribution on a sub-half-millimeter resolution [22]. Images were reconstructed using previously described methods [23], and compensated for distance dependent sensitivity and blurring of the projection of the pinholes during reconstruction through point source based system calibration [24]. The images were registered using an automated registration programme, using set reference points/bed positions. From the SPECT images, the dose distribution could be calculated as described by the MIRD S-voxel method [25]. The MCNPX 2.5.0 Monte Carlo Code [26] was used to estimate the ^166^Ho dose convolution kernel. For each voxel in a 15.375 mm^3^ cube of tissue material (ρ = 1.06 g cm^−1^) the absorbed dose was calculated as a result of the ^166^Ho source uniformly distributed in the center voxel of the cube. The voxel size chosen was 375 µm (isotropic), equal to the voxel size of the reconstructed SPECT images. Dose maps were calculated by convolution of the SPECT images with a simulated ^166^Ho dose kernel. MRI was performed on a 4.7 T horizontal bore small animal scanner (Agilent, UK). Images were acquired using a multi-slice gradient echo MR sequence with an echo time of 3.0 msec and repetition time of 242 msec, a field of view (FOV) of 64×32 mm^2^, a scan matrix of 256×128 with 38 slices resulting in a voxel size of 0.25×0.25×0.5 mm^3^, eight signal averages and a 25° flip angle.

### Histopathologic analysis

Tumor-bearing and contralateral kidney, liver, spleen and heart/lung were weighed and radioactivity was measured in a dose calibrator for qualitative purposes. Quantification of holmium was performed by inductively coupled plasma mass spectrometry. Tumor size was measured using digital callipers and tumor volumes were calculated using the equation V = (A×0.5)×B^2^ (A = largest diameter, B = smallest diameter). Tumor-bearing kidneys were processed and embedded in paraffin. A haematoxylin and eosin (HE) section of each kidney was evaluated for glomerular, tubular and vascular changes and inflammation of the irradiated tumor and surrounding renal parenchyma, as previously described [27], [28].

### Inductively coupled plasma mass spectrometry

Inductively coupled plasma mass spectrometry (ICP MS) was performed to determine the holmium content in the organs used for histopathologic analysis after NaCl or ^166^HoAcAcMS treatment. Paraffin was removed from embedded tissue by gentle heating. The organs were digested in 1 mL *aqua regia* (concentrated nitric, perchloric, and sulphuric acid in a ratio 4∶1∶1) under heating. After destruction, all samples were passed through cotton gauze to remove the insoluble paraffin residue. The samples were diluted in 2% nitric acid, and measured on a Varian 820 MS (Varian, the Netherlands), with a detection limit of 0.1 nanogram holmium mL^−1^ using standard holmium reference material (CertiPUR Holmium ICP Standard (traceable to SRM from NIST), Merck, Darmstadt, Germany).

## Results

### Microsphere administration

MS had a smooth surface, both before and after neutron irradiation and holmium content of the MS was 45% (w/w). The size distribution was not affected by neutron irradiation, and the specific activity of the ^166^HoAcAcMS was 10 MBq mg^−1^ corresponding with 22 Bq ng^−1^ holmium at the time of delivery. ^166^HoAcAcMS were successfully administered to 24 Renca tumor-bearing Balb/C mice. As a control, 36 (n = 3–7 mice per time point) tumor-bearing animals received 10 µl of saline. The mean tumor diameter at the time of treatment was 5.6 mm±1.6 mm. The average administered dose was 2.7 MBq±1.2 MBq (corresponding to 270±120 µg ^166^HoAcAcMS). Hence the detection limit expressed as percentage of the injected dose of holmium was 0.9×10^−4^%.

### Efficacy

**Figure 1:**
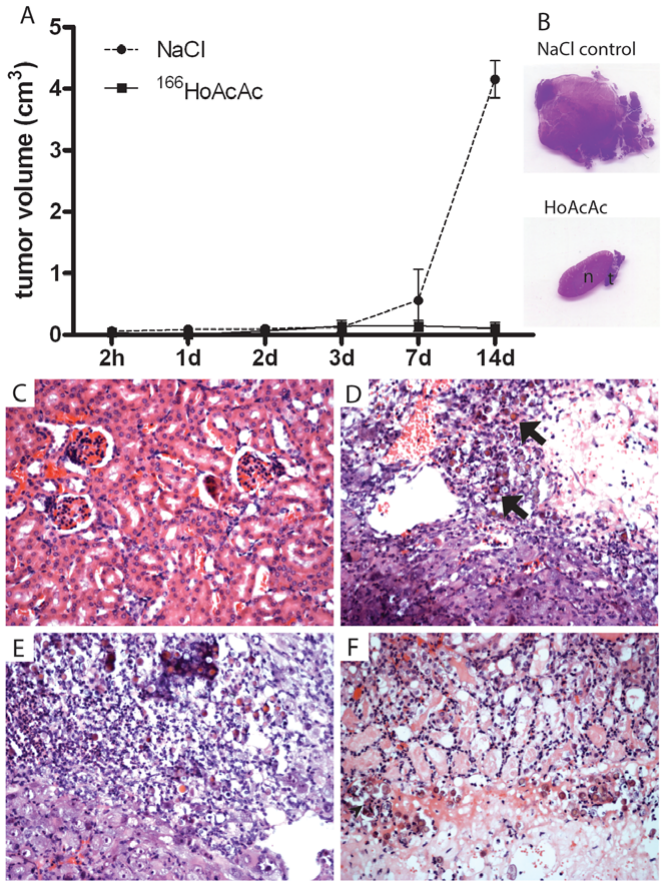
Intratumoral injection of ^166^HoAcAcMS is an effective, minimally invasive procedure in kindney cancer. (**A**) Tumor volume at different time points after treatment. The solid line represents the tumor volume of the ^166^HoAcAcMS group. The dashed line represents the tumor volume of the saline control group. (**B**) HE staining of kidney and tumor tissue 2 weeks after ^166^HoAcAcMS treatment (n indicates normal kidney, t indicates tumor.) (**C**) HE staining (20×) of renal parenchyma outside the radiated region 2 weeks following ^166^HoAcAcMS administration, showing no glomerular, tubular or vascular alterations. (**D**) HE staining (20×) of irradiated tumor 1 day following ^166^HoAcAcMS administration. HoAcAcMS are present as focal intratumoral deposits (arrows). At the site of injection, tumor necrosis and cell death is visible. (**E**) HE staining of irradiated tumor (20×) 2 days following ^166^HoAcAcMS administration. Inflammatory cells are present at the radiated area. (**F**) HE staining of irradiated tumor (20×) 1 week after ^166^HoAcAcMS administration. Grade 3 radiation damage [27] is only visible in renal parenchyma directly surrounding the tumor.

Neither discomfort nor aberrant behaviour was observed in these mice. The bodyweight of mice in the ^166^HoAcAcMS (22.2±1.8 g) and saline group (22.1±1.2 g) remained constant throughout the experiment. The efficacy of ^166^HoAcAcMS after intratumoral injection is depicted in Figure 1A. The tumor volume in the saline control group increased from 0.12±0.03 cm^3^ (day 3 after treatment) to 4.15±0.3 cm^3^ two weeks post injection. Importantly, the tumor volume in the ^166^HoAcAcMS group remained constant from 0.14±0.01 cm^3^ at three days post injection to 0.10±0.01 cm^3^ after two weeks. ICP MS analysis showed that 72.9% (33.1–83.6) (median (IQR)) of the total holmium measured was detected in the tumors of mice that received ^166^HoAcAcMS. In 15 mice in the holmium group 16.9% (IQR 2.6–52.5) holmium was found in the lungs, most likely due to the inadvertent delivery of ^166^HoAcAcMS in a blood vessel in or around the tumor [14].

### Histology

^166^HoAcAcMS were present as focal intratumoral deposits (Figure 1D). Tumor necrosis was visible at the site of injection from 24 h following ^166^HoAcAcMS administration (Figure 1D), inflammatory cells were visible within 48 h (Figure 1E). Grade 3 radiation damage [27] was only visible in renal parenchyma directly surrounding the tumor 2 weeks after treatment (Figure 1F). In all cases, renal parenchyma outside the radiated region showed no glomerular, tubular or vascular alterations (Figure 1A).

### Imaging

**Figure 2:**
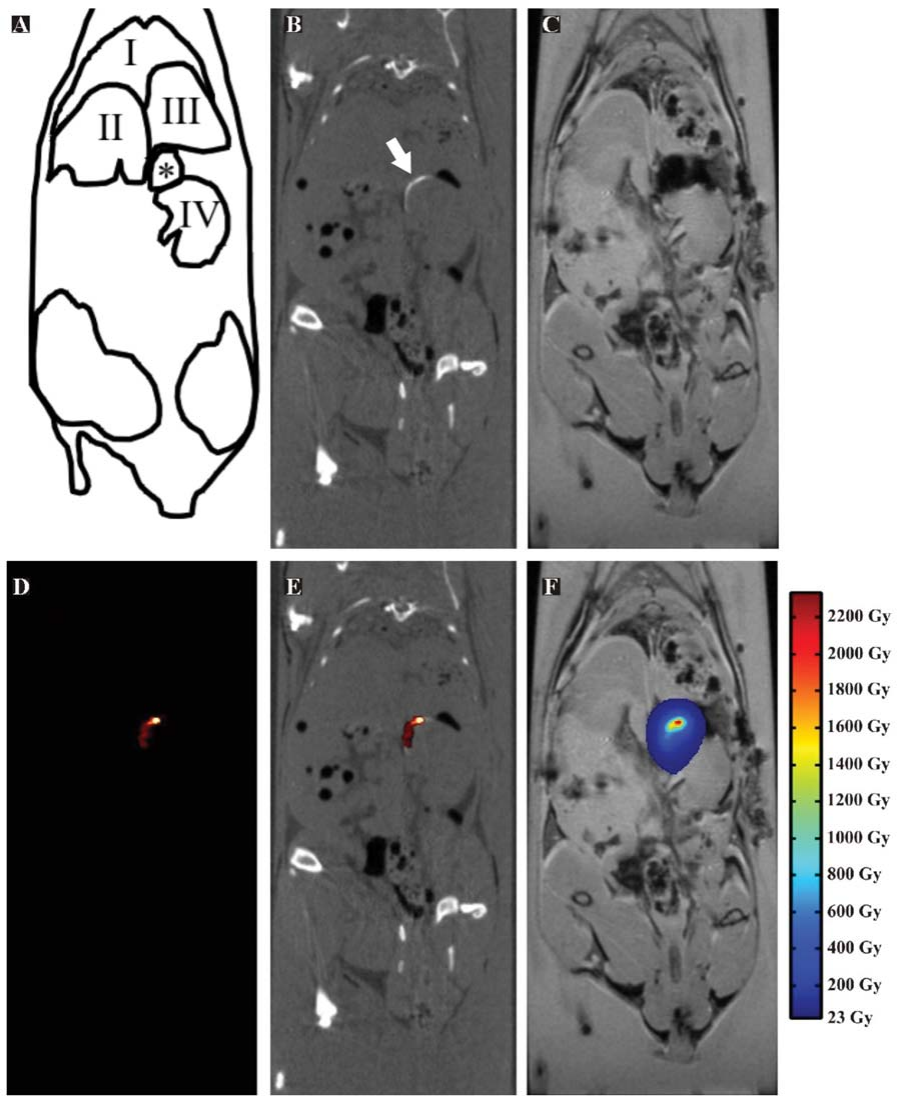
An example of the multimodality imaging characteristics of intratumorally administered ^166^HoAcAcMS. Images are acquired immediately following administration of the MS. (**A**) A schematic representation of the mouse anatomy (I: Lungs; II: Liver; III: Stomach; IV: Kidney; *: Artefact caused by holmium on MRI image 3C) (**B**) CT image, depicting the HoAcAcMS in the kidney area as a white area (arrow). (**C**) MR image showing the soft tissue detail of this imaging modality and the deposition of HoAcAcMS as a dark spot. (**D**) SPECT image, showing a selective visualisation of radioactive MS in the kidney area. (**E**) Fused SPECT and CT image. (**F**) MRI image fused with the dose map, showing the absorbed dose distribution in the kidney area.

**Figure 3:**
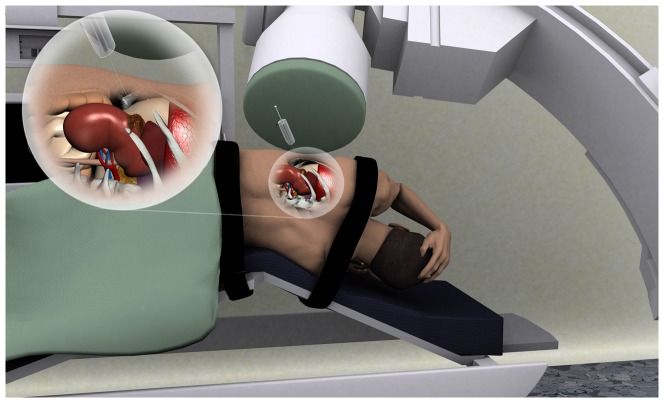
Schematic drawing showing the percutaneous approach to ^166^HoAcAcMS administration. The patient is placed in lateral position in an angiography suite. The ^166^HoAcAcMS are administered intratumorally by a shielded syringe under real-time CT guidance.

The powerful multimodality imaging characteristics of holmium were demonstrated with CT, SPECT and MRI in mice that were terminated immediately following intratumoral administration of ^166^HoAcAcMS (Figure 2A to D). CT imaging revealed deposits of ^166^HoAcAcMS (approximately 300 µg, corresponding to 2.7 MBq ^166^Ho) in the kidney area. Substantial accumulation of particles was seen at the site of injection (Figure 2B). ^166^HoAcAcMS were clearly visualized using SPECT imaging and the SPECT images served as a template for the construction of a dose map (Figure 2E). The dose map shows a selective deposition of therapeutic beta particles at the site of injection, leading to a local tumor absorbed dose in excess of 2200 Gy. The average tumor dose was calculated using the equation as postulated by Vente et al.: Dose (Gy) = (Dosage administered (MBq) ×15.87 mJ MBq^−1^)/tumor weight (g) [29]. The calculated average tumor dose in this study was 323 Gy. The radiation dose to the healthy tissue was below 23 Gy in the largest part of the kidney and surrounding organs (Figure 2F). For a more detailed anatomical depiction of the soft tissue, MR images were acquired. On T_2_* weighted MRI scans, holmium causes a rapid signal decay due to the paramagnetic nature of this element. Consequently, holmium appears as blackening on T_2_* weighted images. As can be seen in Figure 3, holmium is clearly visible as a dark spot in the upper side of the kidney, where it was administered in the tumor. By combining the sensitivity and high quantitative accuracy of SPECT imaging [30], [31], [32] with the soft tissue imaging of MRI, the ^166^HoAcAcMS therapy can accurately be evaluated to ensure complete tumor ablation.

## Discussion

The feasibility and efficacy of ^166^HoAcAcMS as a minimally invasive treatment was assessed in a mouse model for kidney cancer. Importantly, the intratumoral administration of 2.7 MBq ^166^HoAcAcMS arrested tumor growth, which was apparent one week post-administration when approximately 95% of the ionizing radiation dose has been delivered. Assuming all beta energy is deposited in the tumor, the calculated average tumor absorbed dose was 323 Gy, with local doses as high as 2200 Gy. The average tumor absorbed dose is approximately 4–5 fold higher than the 60–80 Gy absorbed dose used in EBRT as a value sufficient for complete tumor kill. Despite the high calculated absorbed dose, the localized nature of the radiation dose considerably reduces unwanted damage of surrounding tissues. The radiation dose to the healthy tissue was below 23 Gy in the largest part of the kidney and surrounding organs. 23 Gy is the maximum tolerance dose for uniform kidney irradiation [33]. Given the maximum penetration depth of the emitted beta particles (8 mm) and the larger size of the kidney, the radiation dose to healthy parenchyma is expected to be negligible when translating to the clinical situation.

In addition to efficacy, the feasibility of multimodality imaging of ^166^HoAcAcMS was demonstrated. Deposits of approximately 2.7 MBq or 270 micrograms of ^166^HoAcAcMS were clearly visible on SPECT, or CT and MRI. ^166^HoAcAcMS can therefore be regarded as a true multimodality imaging agent. The specificity of SPECT was superior to that of both CT and MRI. In this study CT imaging was used to determine the distribution of ^166^HoAcAcMS, since the low amounts of activity administered result in a long acquisition time with SPECT (6 minutes for CT versus 45 minutes SPECT). However, in patients faster SPECT image acquisition will be possible, as the amount of activity will be 20- to 100-fold higher. Furthermore, by combining the sensitivity and high quantitative accuracy of SPECT imaging [30], [31], [32] with the soft tissue imaging of MRI, the ^166^HoAcAcMS therapy could accurately be evaluated to ensure complete tumor ablation during the procedure.

The use of radioactive microspheres in the treatment of an experimental tumor model was successful, and results are very promising. Although the orthotopic mouse model resembles the human situation in many aspects, a number of points need to be addressed before intratumoral administration of ^166^HoAcAcMS could be routinely applied in humans. First of all, toxicity studies should be performed in humans. The *in vivo* stability of the HoAcAcMS was assessed in a tumor bearing rabbit model, which showed the stability of the HoAcAcMS for up to one month, and no toxicity was observed [21]. Radioactive holmium poly(L-lactic acid) particles of 20–50 µm for intra-arterial application have been evaluated for biodistribution, efficacy and toxicity in rats, rabbits and pigs, and have shown their stability and safety [29], [34], a phase-I study of patients with liver metastases has recently been performed [35], [36]. Similar toxicity experiments will need to be performed for the ^166^HoAcAcMS described here prior to initiating a clinical trial.

Tumors in study ranged from 4 to 8 mm at the time of treatment, which is similar to the penetration range of the beta particles in tissue. It is expected that for nephron-sparing treatment of tumors smaller than 4 cm, 4 to 8 injections with ^166^HoAcAcMS are required for an effective eradication. This is comparable to conventional minimally invasive ablative treatments that often need multiple probes to achieve complete tumor kill [37]. The advantage of radionuclide therapy compared to thermal ablative minimally invasive techniques is the prolonged delivery of energy to the tumor tissue, due to the half life. Using multiple deposits of ^166^HoAcAcMS, an accurate delivery of the dose can be given independent of shape and size of the tumor. As observed, an average tumor dose of 323 Gy was obtained with one deposit. When positioning the deposits within 1 cm apart it is expected that complete tumor kill can be achieved. However, more research is warranted to further investigate the relationship between deposit localisation and tumor kill in larger tumors.

A technical problem we encountered was the delivery of approximately 50% of the intended dose (5 MBq), possibly due to adsorption of radioactive MS onto the syringe wall and dosage errors due to the small injected volume. When treating patients, the administered volume is higher, resulting in a smaller difference between aimed and actual administered dose. Furthermore, varying concentrations of holmium were found in the lungs of several mice. Tumor vascularity as well as particle size is an important factor for the fate of the radioactivity [38]. Although the presence of holmium in the lungs is likely due to the inadvertent vascular delivery of the MS during the intratumoral injections [14] and with a size of 12.5 µm diffusion within the tissue is less likely, a future study should be performed to examine these two factors specifically. Lung uptake was not observed when administering ^166^HoAcAcMS percutaneously under ultrasound guidance in hypervascular feline liver tumors [38]. Ultrasound guidance not only helps to distinguish healthy tissue from tumorous tissue, it also visualises tumor vasculature. Unfortunately, percutaneous ultrasound guided administration was not feasible in this model. Nevertheless, in patients the procedure should be performed percutaneously under CT or ultrasound guidance, to ensure accurate delivery and avoid inadvertent vascular delivery. An illustration of a CT guided approach is shown in Figure 3. A new method that could be used is the multiplanar Global Positioning System-like technology [39]. A minimally invasive approach would make intratumoral administration of ^166^HoAcAcMS suitable for treatment of patients for whom surgical resection is considered inappropriate.

## Conclusions

The present study demonstrates that intratumoral administration of ^166^HoAcAcMS is a promising novel minimally invasive treatment of kidney cancer. Tumor growth was arrested and no signs of radiation damage outside the treatment zone were observed. Importantly, multimodality imaging including CT, SPECT and MRI of small amounts of ^166^HoAcAcMS was feasible. This will lead to an improved therapy evaluation and follow-up and provides a fundamental advantage over current therapies.
